# Liver Metastasectomy in Anal Squamous Cell Carcinoma: The Mayo Clinic Experience

**DOI:** 10.3390/curroncol33030157

**Published:** 2026-03-10

**Authors:** Noah Takacs, Conor D. J. O’Donnell, Nguyen Tran, Krishan Jethwa, Thomas Atwell, Patrick Starlinger, Zhaohui Jin

**Affiliations:** 1 Department of Internal Medicine, Mayo Clinic, Rochester, MN 55905, USA; 2 Department of Internal Medicine, Mayo Clinic, Jacksonville, FL 32066, USA; 3 Department of Medical Oncology, Mayo Clinic, Rochester, MN 55905, USA; 4 Department of Radiation Oncology, Mayo Clinic, Rochester, MN 55905, USA; 5 Department of Radiology, Mayo Clinic, Rochester, MN 55905, USA; 6 Department of Surgery, Mayo Clinic, Rochester, MN 55905, USA

**Keywords:** anal squamous cell carcinoma, liver metastases, metastasectomy, liver-directed therapy, oligometastatic disease

## Abstract

Background: Metastatic squamous cell carcinoma of the anus (SCCA) carries a poor prognosis, with systemic therapy remaining the standard of care. While metastasis-directed therapy improves outcomes in select gastrointestinal malignancies, the role of liver-directed intervention in metastatic SCCA remains undefined. We evaluated clinicopathologic features and oncologic outcomes of patients with liver-limited metastatic SCCA treated with curative-intent hepatic local therapy at a tertiary academic center. Methods: We conducted a retrospective cohort study of adults with histologically confirmed SCCA and liver-only metastatic disease who underwent curative-intent hepatic resection or ablation at Mayo Clinic between 1993 and 2023. Patients with extrahepatic disease or incomplete records were excluded. Demographic, tumor, treatment, and outcomes data were abstracted from electronic medical records. Overall survival (OS) and disease-free survival (DFS) were estimated using the Kaplan–Meier method. Prognostic factors were assessed using univariate Cox proportional hazards models. Results: Twenty-five patients met inclusion criteria. Median age was 56.7 years, and 92% were female. Most patients had metachronous metastases (76%), a single hepatic lesion (56%), and unilobar disease (76%). Pre-intervention systemic therapy was administered in 52% of patients, with radiographic complete or partial responses observed in all treated patients. Liver-directed therapy consisted of surgical resection in 80% and thermal ablation in 20%. Among surgical patients, 90% achieved microscopically negative margins. With a median follow-up of 22 months, disease recurrence occurred in 80% of patients, most commonly within the liver. Median DFS was 7.27 months. Median OS from the date of liver-directed therapy was 51.3 months. On univariate analysis, poorly differentiated tumor histology was associated with inferior OS (hazard ratio 4.67, *p* = 0.018). No other clinicopathologic variables were significantly associated with DFS or OS. Conclusions: In this single-institution experience, carefully selected patients with liver-limited metastatic SCCA undergoing curative-intent hepatic-directed therapy achieved prolonged overall survival, substantially exceeding historical outcomes with systemic therapy alone. Despite frequent recurrence, the observed median OS exceeding four years supports consideration of liver-directed therapy within a multidisciplinary framework for patients demonstrating favorable disease biology and response to systemic treatment. Prospective studies are needed to better define patient selection and optimal integration of local and systemic therapies in the modern treatment era.

## Simple Summary

Metastatic squamous cell carcinoma of the anus remains a rare disease with poor prognosis, and evidence guiding the management of liver-limited metastatic disease is sparse. While platinum-based chemotherapy with immunotherapy represents the current standard of care, long-term survival remains uncommon. The role of metastasis-directed therapy in this population is not well defined, with existing data largely restricted to small series. In this single-institution retrospective cohort study spanning three decades, we report outcomes for 25 patients with liver-only metastatic anal squamous cell carcinoma who underwent curative-intent liver-directed therapy. We demonstrate a median overall survival of 51.3 months, substantially exceeding historical outcomes reported with systemic therapy alone and prior multi-institutional surgical series. Our data suggested that liver-directed therapy may be associated with favorable outcomes in highly selected patients.

## 1. Introduction

Anal cancer is a rare gastrointestinal malignancy, with approximately 10,930 new cases (3560 male and 7370 female) and 2030 cancer-related deaths estimated in the United States in 2025, accounting for approximately 3% of digestive system cancers [1]. Anal cancer can arise from any of the three types of mucosa in the anal region (glandular, transitional, and squamous mucosa). Squamous cell carcinoma of the anus (SCCA) is the most common histologic subtype, accounting for 85–90% of all anal cancers with a female dominance [2].

The incidence of invasive SCCA increased at an average annual percentage change (AAPC) of 2.9% from 1992 to 2001 and 2.7% from 2001 to 2015 [3,4]. Further analysis from the Surveillance, Epidemiology, and End Results (SEER) program demonstrated a similar AAPC of 2.1% in the overall population, with a higher rate of 2.8% among individuals aged ≥ 50 years [5]. Among patients aged ≥ 50 years, females had a higher AAPC of 3.3% compared with 2.1% in males, and the APC was highest in the 55–59-year age group (4.3%) [5]. Meanwhile, mortality from SCCA has also increased steadily over recent decades, with an average AAPC of 3.1% [3].

The prognosis of SCCA is largely determined by the size of the primary tumor and the presence of locoregional lymph node metastases [6,7,8,9]. A recent analysis of the National Cancer Database (NCDB, 2004–2020) including 76,830 patients with SCCA reported that 50.4% presented with stage I/II disease, 6.5% presented with synchronous metastatic disease, and approximately 29% had node-positive disease based on meta-analytic data [10,11,12]. Patients with locoregional SCCA typically achieve favorable outcomes following definitive chemoradiotherapy, with a reported 5-year disease-free survival of 78% [13]. However, patients presenting with metastatic SCCA have substantially worse outcomes, with a 5-year relative survival rate of 36% [14]. The liver, lungs, and extrapelvic lymph nodes are the most frequently involved metastatic sites [15,16].

Systemic therapy represents the cornerstone of management for metastatic SCCA. A retrospective study including 53 patients with metastatic SCCA demonstrated activity of the FOLFCIS regimen (fluorouracil, leucovorin, and cisplatin), with a response rate (RR) of 48%, median progression-free survival (PFS) of 7.1 months, and median overall survival (OS) of 22.1 months, supporting its use in the first-line setting prior to the InterAACT study [17].

The phase II InterAACT trial enrolled 91 patients with advanced SCCA and randomized them to receive fluorouracil/cisplatin (5-FU 1000 mg/m^2^ on days 1–4 and cisplatin 60 mg/m^2^ on day 1, every 21 days) or carboplatin/paclitaxel (carboplatin AUC 5 on day 1 and paclitaxel 80 mg/m^2^ on days 1, 8, and 15 every 28 days) [18]. The primary endpoint was objective response rate (ORR). ORR was 57% (95% CI, 39.4–73.7%) in the cisplatin/5-FU arm and 59% (95% CI, 42.1–74.4%) in the carboplatin/paclitaxel arm. Median PFS and OS were 5.7 and 12.3 months, respectively, for the cisplatin/5-FU arm compared with 8.1 and 20 months for the carboplatin/paclitaxel arm (hazard ratio [HR] 1.27, *p* = 0.375 for PFS; HR 1.78, *p* = 0.059 for OS). Although median PFS and OS were numerically higher in the carboplatin/paclitaxel arm, these differences did not reach statistical significance, likely due to the limited sample size and reduced statistical power. However, grade ≥ 3 adverse events strongly favored the carboplatin/paclitaxel arm (serious adverse event rate 62% in the cisplatin/5-FU arm vs. 36% in the carboplatin/paclitaxel arm). Given its more favorable toxicity profile and numerical improvement in survival outcomes, carboplatin/paclitaxel became the preferred first-line chemotherapy regimen until the emergence of chemoimmunotherapy data from the InterAACT-2 study.

Immunotherapy, particularly anti–PD-1 inhibitors, has demonstrated modest activity in later-line treatment of advanced SCCA, with reported response rates ranging from 11 to 24% [19,20,21]. The POD1UM-202 study was a phase II trial enrolling 94 patients with previously treated advanced or metastatic SCCA [18]. Patients received retifanlimab, a humanized monoclonal antibody targeting PD-1 (500 mg every 4 weeks), with overall response rate (ORR) as the primary endpoint. At a median follow-up of 7.1 months, ORR was 13.8%, with a disease control rate (DCR) of 48.9%.

The InterAACT-2 (POD1UM-303) study is an international, multicenter, phase III trial designed based on the POD1UM-202 study [14]. The study enrolled 308 treatment-naïve patients with locally recurrent or metastatic SCCA and randomized them (1:1) to carboplatin/paclitaxel with retifanlimab versus placebo for up to one year. Patients in the placebo arm were allowed to cross over after disease progression. The primary endpoint was PFS. Median PFS was 9.3 months (95% CI, 7.5–11.3) in the retifanlimab group compared with 7.4 months (95% CI, 7.1–7.7) in the placebo group (HR = 0.63 [95% CI 0.47–0.84]; one-sided *p* = 0.0006). The U.S. Food and Drug Administration approved retifanlimab in combination with carboplatin and paclitaxel as first-line treatment for advanced SCCA, as well as retifanlimab monotherapy in the appropriate setting, on 15 May 2025. Retifanlimab combined with carboplatin/paclitaxel is now the standard first-line treatment for advanced SCCA. However, data beyond first-line platinum-based chemoimmunotherapy remain limited.

As demonstrated in colorectal and neuroendocrine malignancies, local treatment of metastatic sites in highly selected patients with oligometastatic disease may offer improved disease control and prolonged survival. However, the role of metastasis-directed therapy, including metastasectomy, in SCCA remains undefined, and published data are largely limited to small case series and database analyses [22,23]. Although long-term survivors have been identified, recurrence remains common, and optimal patient selection criteria for surgery are uncertain.

Given the rarity of liver-limited metastatic SCCA and the paucity of detailed outcomes data, further characterization of this patient population is warranted. The present study describes the clinicopathologic features, treatment approaches, and oncologic outcomes of patients with liver-only metastatic SCCA who underwent curative-intent local therapy at a single tertiary academic institution over a 30-year period.

## 2. Methods

### 2.1. Study Design and Patient Selection

This was a single-institution retrospective cohort study conducted at the Mayo Clinic and approved by the Mayo Clinic Institutional Review Board (IRB number 23-010177). The requirement for informed consent was waived. All patients aged 18 years or older with a diagnosis of histologically confirmed SCCA and liver-only metastatic disease who underwent curative-intent local therapy between 1 January 1993, and 18 October 2023, were eligible for inclusion. Patients were identified through a comprehensive search of the prospectively collected Mayo Advanced Text Explorer (ATE) database and electronic medical records were reviewed with detailed data extraction. Patients with extrahepatic metastases at diagnosis, incomplete medical records, or a concurrent active malignancy (other than prostate cancer, non-muscle invasive bladder cancer, or non-melanomatous skin cancer) were excluded.

### 2.2. Data Collection

Demographic, clinicopathologic, treatment, and outcomes data were abstracted from the electronic medical record by trained investigators. Recorded variables included patient demographics (date of birth, sex, age at diagnosis, performance status, smoking history, and HPV and PD-L1 status); tumor characteristics (histology, differentiation, T, N, and M stage, and inguinal lymph-node involvement); and details of primary disease management (primary tumor resection, response to locoregional therapy, and locoregional recurrence). Characteristics of liver metastases, including number, maximal diameter, and lobar distribution, were abstracted from radiologic and operative reports. Systemic therapy details encompassed pre- and postoperative regimens, duration, and radiographic response of liver lesions. Chemotherapy regimens were categorized by drug class with start and stop dates recorded. Systemic therapy duration prior to liver-directed treatment was dichotimized as ≤12 months versus >12 months. The radiographic response of hepatic disease to preoperative therapy was categorized as complete, partial, stable, or progressive disease. Liver-directed interventions were classified as hepatic resection or ablation (either radiofrequency or microwave). The type of resection (wedge, segmentectomy, hemihepatectomy, or extended hepatectomy) and surgical margin status were recorded. Margin status was categorized as microscopically negative, close (<1 mm), or microscopically positive.

### 2.3. Follow-Up and Outcome Assessment

All patients were followed longitudinally through Mayo Clinic records. Recurrence was defined as radiographic or histologic evidence of new or progressive disease after completion of local therapy. Overall survival (OS) was measured from the date of liver-directed therapy to the date of death. Disease-free survival (DFS) was measured from the date of liver-directed therapy to the date of recurrence or death. Patients alive without recurrence were censored at the date of last contact.

### 2.4. Statistical Analysis

Descriptive statistics were used to summarize patient and treatment characteristics. Continuous variables were expressed as medians, and categorical variables as counts and percentages. Survival distributions for OS and DFS were estimated using the Kaplan–Meier method. Prognostic factors associated with OS and DFS were evaluated using univariate Cox proportional hazards models. Hazard ratios (HR) and 95% confidence intervals (CI) were calculated. Exploratory subgroup analyses were performed comparing outcomes by treatment modality, timing of metastasis (synchronous vs. metachronous), hepatic disease distribution (unilobar vs. bilobar), among others. Statistical analyses were performed using BlueSky Statistics software (version number 10.3.4). A two-sided *p*-value < 0.05 was considered statistically significant.

## 3. Results

### 3.1. Clinicopathologic Characteristics

The clinical characteristics of the cohort are summarized in Table 1. Twenty-five patients met inclusion criteria and underwent curative-intent local management of liver-limited metastatic ASCC. The median age was 56.7 (32.9–77.2) years, and the cohort was predominantly female (92%). No patients had known HIV infection. HPV positivity was present in 80% of patients evaluated. Most patients presented with metachronous hepatic metastases (76%). With respect to hepatic disease burden, 56% harbored a single metastatic lesion and 76% had unilobar involvement.

### 3.2. Treatment Details

Pre-intervention systemic therapy for metastatic disease was administered in 52% of patients (median duration, 13.2 months), with the most common regimen being a combination of carboplatin and a taxane. Of those treated, 69% had a complete response and 31% had a partial response. Definitive local treatment of hepatic disease consisted of surgical resection in 80% of patients and thermal ablation in 20%. Among patients who underwent a surgical resection (*n* = 20), the extent of resection included hemihepatectomy in 20% and segmentectomy in 80%. Pathologic margin assessment demonstrated margins that were microscopically negative in 90% and close (<1 mm) in 10%, with no microscopically positive margins observed (Table 2).

### 3.3. Patterns of Survival and Recurrence

**Figure 1:**
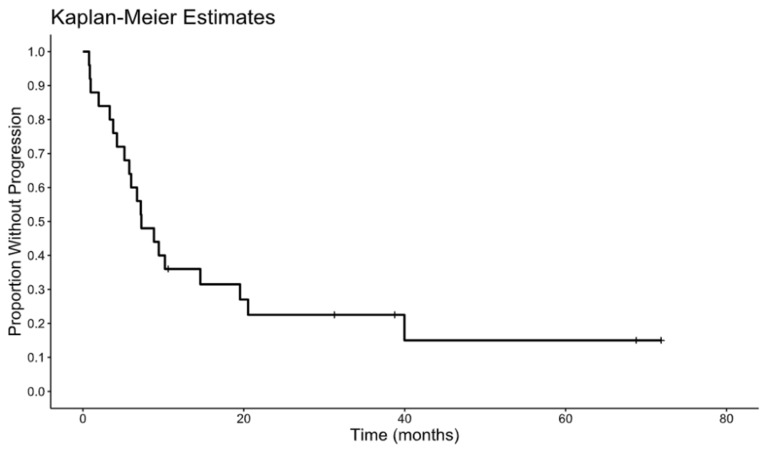
Disease-free survival.

**Figure 2:**
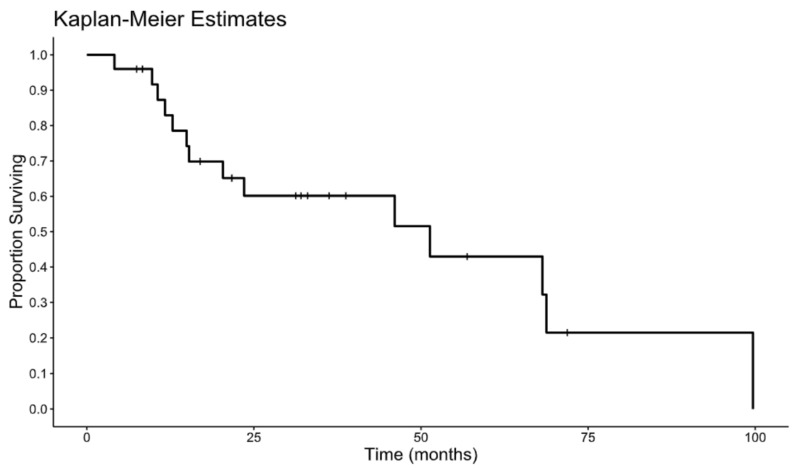
Overall survival.

With a median follow-up of 22 months, 20 of 25 patients (80%) experienced disease recurrence. The liver was the most common site of first recurrence. Among those whose disease recurred, 13 (65%) had intrahepatic recurrence and 7 (35%) had extrahepatic recurrence. The median disease-free survival was 7.27 months. The Kaplan–Meier estimate for DFS is shown in Figure 1. The median overall survival was 51.3 months from the date of surgery. The Kaplan–Meier estimate for OS is depicted in Figure 2. On univariate Cox analyses, poorly differentiated tumor status was associated with significantly worse overall survival outcomes (hazard ratio 4.67, *p* = 0.018). No additional prespecified clinicopathologic features demonstrated statistically meaningful associations with DFS or OS in this cohort. Table 1, Table 2 and Table 3 and Figure 1 and Figure 2 contain the full descriptive statistics, treatment distributions, recurrence patterns, and survival estimates referenced above.

## 4. Discussion

In this single-institution retrospective cohort study, we report long-term outcomes of 25 patients with liver-limited metastatic SCCA who underwent curative-intent liver metastasectomy/ablation. With a median follow-up of 22 months, the median disease-free survival (DFS) was 7.3 months, and the median overall survival (OS) was 51.3 months. These data demonstrate that patients with isolated hepatic-only metastases from SCCA may benefit from liver-directed intervention, achieving prolonged survival that substantially exceeds the survival expected with systemic therapy alone.

In the InterAACT trial, patients treated with first-line carboplatin and paclitaxel achieved a median OS of 20 months while the mOS was 29.2 months in the InterAACT-2/POD1UM303 study at interim analysis [14,21]. In contrast, our surgically managed cohort achieved a median OS exceeding four years, acknowledging that this is a highly selected population. The improved survival in our series may reflect careful patient selection (majority of patients had a single metastatic liver lesion), advances in perioperative care, and modern systemic therapy integration, as well as the exclusive inclusion of patients with a limited number of liver-only metastases of anal origin.

Our study results are consistent with other reports. The multi-institutional analysis by Pawlik et al. reported a median OS of 22.3 months and 5-year survival of 20.5% among patients undergoing hepatic resection for metastatic squamous cell carcinoma [23]. A systemic review conducted in 2025 including 10 studies (total 98 patients) reported that one-year, three-year, and five-year OS rates of 87%, 53% and 38%, respectively [24]. Another retrospective study using the NCDB database (2004–2014) reported that among 2258 patients with metastatic anal cancer, 165 underwent metastasectomy [22]. Among patients who underwent liver metastasectomy, the median OS was 34 months compared to 16 months in those who did not undergo liver metastasectomy (*p* < 0.0001).

However, after curative-intent liver metastasectomy, disease recurrence remains common, with liver being the most frequent site of recurrence. In our case series, 80% of patients experienced disease recurrence, most frequently within the liver. This pattern mirrors prior reports in which intrahepatic relapse predominated following resection of SCCA metastases [23,25]. Among patients undergoing hepatic resection, margin-negative (R0) resection was achieved in 90% of cases. All resected specimens contained viable tumors, confirming that radiographic responses may underestimate residual disease burden. The predominance of segmentectomy or limited hepatectomy in our cohort suggests that parenchymal-sparing techniques are feasible in this setting.

Within our case series, 13 patients received preoperative systemic treatment. We did not capture the specific rationale for omission of preoperative chemotherapy in the remaining patients; however, this likely reflects temporal treatment differences and the predominance of delayed metachronous limited liver metastatic disease (19 of 25 cases developed metachronous liver metastatic disease). Importantly, among patients who received preoperative systemic treatment, none of them had disease progression prior to surgical intervention, emphasizing that surgical consideration was limited to those demonstrating disease control or response to systemic therapy. These findings support a paradigm in which favorable biology and chemosensitivity, rather than radiographic extent alone, are critical for patient selection for liver oligometastasis-directed treatment [26].

On univariate analysis, poor histologic differentiation was associated with inferior OS, consistent with prior reports linking tumor grade to more aggressive biology. Other clinicopathologic variables, including timing of metastases (synchronous vs. metachronous), number of lesions, and lobar distribution, did not significantly influence outcomes. However, our sample size of 25 patients significantly limits statistical power and our ability to detect true differences. Furthermore, as our study included only patients who underwent metastasis-directed therapy, all medical history, disease natural history, and anatomic criterion involved in patient selection for local therapy likely selected for a favorable cohort, and thus the generalizability of conclusions may not apply in all situations of oligo- or poly-metastatic SCCA. The high proportion of HPV-positive and immunocompetent patients in our cohort may have contributed to the relatively favorable long-term survival, as HPV-related SCCA is known to exhibit distinct immunologic responsiveness. A large meta-analysis including 693 individual patient data demonstrated that patients with HPV positive anal cancer had superior OS compared to those HPV negative tumors (HR 0.26, 95% CI: 0.14–0.50) and another systemic review with meta-analysis also showed a hazard ratio for OS of 0.54 favoring HPV-DNA positive anal cancer [27,28].

Within our case series, we also observed that at least 10 patients developed metachronous liver metastases during surveillance while their primary tumors remained in clinical complete response (CCR). This suggests that occult liver metastatic disease may have been present long before the liver lesions were detected radiographically. This raises the question of the potential role of circulating tumor DNA (ctDNA) testing for minimal or molecular residual disease (MRD) detection in SCCA. ctDNA testing has high sensitivity for SCCA, with baseline detection rate of 88–91% across all stages [29,30]. Both tumor-informed personalized ctDNA assays and HPV-specific assays may potentially be used, given approximately 90% of SCCA are HPV-related. Although there is no direct comparison between these two approaches, tumor-informed ctDNA assay may offer broader sensitivity, as it can also cover these HPV negative SCCA [31,32]. Studies have demonstrated promising prognostic value of ctDNA in SCCA. Among patients with stage I-III SCCA, any ctDNA positivity after definitive treatment is associated with poor clinical outcomes (HR for DFS 28, *p* = 0.005), and ctDNA positivity often precedes radiographic evidence of disease recurrence [30]. Although further evidence is needed to determine whether early detection and early intervention for metastatic disease improve clinical outcomes, studies in other tumor types, particularly in colorectal cancer and bladder cancer, suggested potentially beneficial [33,34,35]. We have limited cases in this series who underwent tumor-informed ctDNA MRD monitoring, and we were able to detect early recurrent disease based the positive ctDNA results, prompting additional image studies and shorter surveillance intervals.

Limitations of this study include its retrospective design and limited cohort size, which precluded multivariate modeling. Additionally, our study has inherent selection bias toward patients with favorable disease biology and performance status, as patients who progressed on chemotherapy were not offered metastasectomy. Despite these constraints, to our knowledge this represents one of the largest single-institution experiences of hepatic-directed therapy for metastatic SCCA, providing contemporary real-world data to inform clinical decision-making and future trial design.

## 5. Conclusions

Our findings support the consideration of liver-directed therapy within a multidisciplinary framework for patients with isolated hepatic metastases from SCCA who achieve disease control with systemic therapy. The observed median OS of 51.3 months underscores the potential for durable benefit in this rare clinical context. Nonetheless, recurrence remains common, and prospective studies are required to define the optimal sequencing of systemic and local modalities. Future research should also investigate the ideal treatment regimen following recurrence in these patients. Incorporation of modern systemic agents, including immunotherapy, may further improve outcomes. Additionally, the role of stereotactic body radiation therapy (SBRT) in this setting merits further investigation. Finally, emerging biomarkers such as tumor-informed personalized ctDNA may further inform recurrence risk and identify early recurrent disease that could allow earlier intervention and potentially improved outcomes.

Liver-directed therapy for carefully selected patients with liver-limited metastatic SCCA is associated with favorable long-term survival compared with historical systemic therapy outcomes. Multidisciplinary evaluation remains essential to identify appropriate candidates, and ongoing investigation is warranted to better define the role of metastasectomy in the modern era of combined systemic and local therapy.

## Figures and Tables

**Table 1:** Patient characteristics.

Variable	*n* = 25, *n* (%)
Age at diagnosis	
Median (years), range	56.7 (33–77)
Gender	
Female	23 (92)
Male	2 (8)
ECOG status	
0	9 (36)
1	15 (60)
2	1 (4)
Smoking history	
Yes	13 (52)
No	12 (48)
HPV status	
Positive	12 (48)
Negative	3 (12)
Missing	10 (40)
Tumor histology	
SCC	20 (80)
Basaloid SCC	5 (20)
Tumor differentiation	
Moderately differentiated	7 (28)
Poorly differentiated	12 (48)
Undifferentiated	2 (8)
Missing	4 (16)
T stage of primary tumor	
T1	11 (44)
T2	14 (56)
Nodal status of primary tumor	
Positive	18 (72)
Negative	7 (28)
Number of hepatic metastases (range)	
1	14 (56)
2–3	8 (32)
>3	3 (12)
Median maximal diameter of liver metastases (range), cm	3 (0.9–11.9)
Location of hepatic metastases	
Unilobar	19 (76)
Bilobar	6 (24)

**Table 2:** Treatment details.

Variable	*n* = 25, *n* (%)
Preoperative chemotherapy (*n* = 13)	
Mean preoperative chemotherapy duration (months)	13.2
Preoperative chemotherapy regimen	
Carboplatin/taxane	8 (62)
Cisplatin/5-fluorouracil	4 (31)
Pembrolizumab	1 (8)
Liver metastasis response to preoperative chemotherapy	
Complete response	9 (69)
Partial response	4 (31)
Procedure directed at hepatic metastasis (*n* = 25)	
Liver resection	
Right/left hemi-hepatectomy	4 (16)
Segmentectomy	16 (64)
Thermal ablation	5 (20)
Liver surgical margins (*n* = 20)	
Negative	18 (90)
Close margin	2 (10)
Postoperative chemotherapy (*n* = 7)	
Mean postoperative chemotherapy duration (months)	11.5
Postoperative chemotherapy regimen	
Cisplatin/5-fluorouracil	4 (57)
Carboplatin/nab-paclitaxel	1 (14)
mFOLFOX6	1 (14)
Pembrolizumab	1 (14)

**Table 3:** Prognostic factors.

Prognostic Factor	Disease-Free Survival		Overall Survival	
	Hazard Ratio	95% CI	Hazard Ratio	95% CI
Clinical				
Age > 65	1.17	0.44–3.12	1.27	0.41–4.0
Female gender	1.8	0.25–14.2	0.7	0.09–5.66
ECOG status 1 (vs. 0)	1.46	0.58–3.67	2.16	0.64–7.25
Smoking history	1.6	0.61–4.17	2.64	0.71–9.91
Poorly differentiated status	1.68	0.63–4.48	4.67	1.3–16.7
Hepatic metastasis				
Number of metastases > 1	2.17	0.84–5.62	1.05	0.33–3.34
Bilobar involvement	1.56	0.53–4.55	0.73	0.16–3.43
Treatment details				
Preoperative chemotherapy	0.96	0.39–2.37	0.53	0.16–1.76
Preoperative chemotherapy duration > 12 months	2.05	0.52–8.08	0.99	0.14–7.06
Metastasis response to chemotherapy (CR vs. PR)	0.9	0.23–3.52	0.6	0.06–5.77
Post operative chemotherapy	0.59	0.21–1.64	0.4	0.09–1.81

## Data Availability

The raw data supporting the conclusions of this article will be made available by the authors on request.
